# In this issue: Volume 115, Issue 4, April 2024

**DOI:** 10.1111/cas.16156

**Published:** 2024-04-10

## Blueprints from plane to space: outlook of next‐generation three‐dimensional histopathology

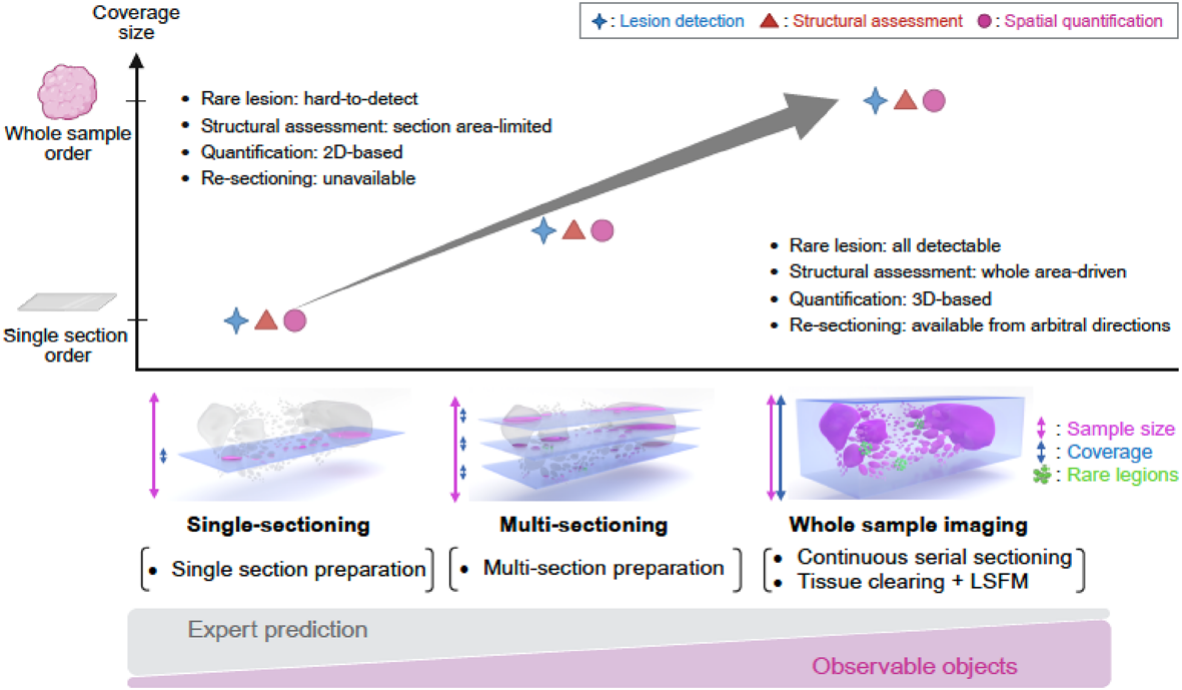

Traditionally, the diagnosis for several diseases is either suspected or confirmed by viewing thin tissue sections under a microscope to spot signature manifestations of a disease, known as clinical histopathology. While this provides a 2D representation of the tissue structure, it does not capture the inherent three‐dimensional complexity, which could provide crucial insight for the diagnosis of diseases presenting intricate microscopic changes that is not apparent in the 2D tissue sections.

Preparing and assessing 2D tissue sections of wide areas becomes time‐consuming, and coupled with low sampling rates, it could lead to errors in reporting. This problem is compounded by the reliance on subjective descriptions and determination of tumor size based on human perception. Incorporating 3D imaging to histopathology can help address these limitations and improve diagnostics.

Already, innovations like serial sectioning, tissue clearing, light‐sheet microscopy, and artificial intelligence‐driven digital image analysis are marking a profound shift in the field. These advancements can offer in‐depth insights into tissue structures, paving the way for improved diagnostic precision and superior patient care.

Although 3D histopathology shows high clinical potential, there are a few challenges related to the time, cost, and image quality. Nevertheless, 3D histopathology signifies a paradigm shift in pathological diagnosis, presenting a transformative approach that synergizes with information science and artificial intelligence. This synergy is essential for overcoming existing challenges, fostering cross‐disciplinary collaboration, and driving innovation within histopathology.

It is important to note that 3D histopathology is positioned not as a replacement, rather as a complement to conventional methods. This integration can contribute towards a deeper understanding of human pathophysiology and better patient care. The transformative potential of 3D histopathology signifies a new era in pathology, where cutting‐edge technologies converge to provide a more holistic and accurate perspective on disease pathology.

https://onlinelibrary.wiley.com/doi/full/10.1111/CAS.16095

## An engineered Accum‐E7 protein‐based vaccine with dual anti‐cervical cancer activity

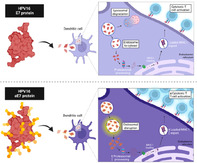

Human papillomavirus (HPV) infections are correlated with genital warts and potential development of cervical, anal, or head and neck cancer. While prophylactic HPV vaccines have demonstrated efficacy in preventing infection, currently available modalities for treating established tumors are limited. This highlights the pressing need for better solutions.

Currently available preventive vaccines against HPV require administration before exposure, rendering them ineffective against existing infections or established cervical cancer. To tackle this issue, scientists have adopted an innovative approach, leveraging the Accum™ technology platform to design the Accum‐E7 (aE7) vaccine. This vaccine offers not only prophylactic defense but also therapeutic action against pre‐existing HPV‐associated tumors.

The researchers demonstrated that prophylactic vaccination with aE7 conferred complete protection against cervical cancer, surpassing the efficacy of traditional E7 protein vaccination. Further, the therapeutic administration of a combination of aE7 and immune checkpoint blockers could effectively control tumor growth when tested in animals with pre‐established tumors. It was also well‐tolerated when tested in mice, showing no adverse side effects even at four‐fold higher doses.

The aE7 vaccine, with its dual prophylactic and therapeutic capabilities, presents a promising candidate for further investigation and clinical testing. The aE7 vaccine is a protein‐based vaccine, with a simplified manufacturing process and better scalability compared to traditional vaccines that employ virus‐like particles. Thus, aE7 marks a significant stride toward addressing the limitations of existing HPV vaccines. The findings call for continued research and clinical exploration, heralding a potential paradigm shift in the fight against HPV‐related malignancies.

https://onlinelibrary.wiley.com/doi/full/10.1111/CAS.16096

## Concurrent targeting of GSK3 and MEK as a therapeutic strategy to treat pancreatic ductal adenocarcinoma

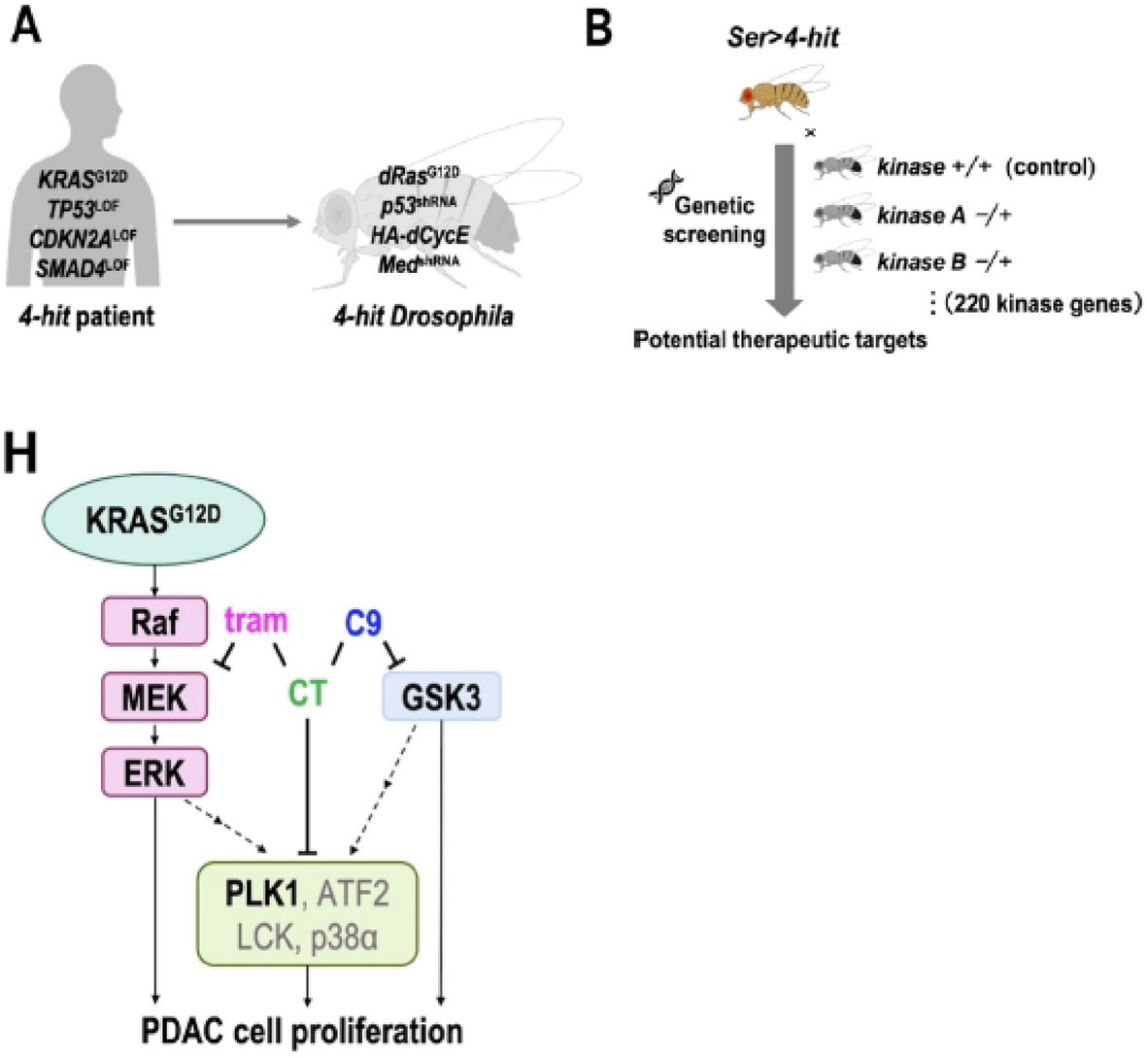

Pancreatic ductal adenocarcinoma (PDAC) is an aggressive form of cancer in the pancreas, with a challenging course of treatment. Common treatments like FOLFIRINOX or gemcitabine have been attempted in the past to mitigate the aggressive nature of PDAC, but existing therapeutic approaches are still limited in their efficacy. These approaches provide only modest relief and may not improve patient outcomes. The complicated biology of PDAC and associated resistance mechanisms highlights an urgent need for novel treatment strategies.

Researchers have now turned to the simple model organism *Drosophila* (fruit flies) for potential answers. In a recent study, scientists utilized a whole‐body genetic screening method in a *Drosophila* model that faithfully mirrors the genetics of PDAC in humans. Upon analysis, they uncovered previously elusive therapeutic targets, focusing on addressing the limitations of existing treatments.

In particular, Glycogen synthase kinase 3 (GSK3) was identified as a pivotal therapeutic target. Upon testing in mice containing human PDAC cells, the combination of an inhibitor of GSK3 with a mitogen‐activated protein kinase (MEK) inhibitor showcased marked efficacy in suppressing PDAC tumor growth. This presents a potential breakthrough in treating the aggressive cancer, with a more nuanced understanding of the underlying mechanisms.

The study contributes to understanding PDAC mechanisms and developing targeted therapies by utilizing a drosophila model in conjunction with mammalian models. In the face of the complexities posed by PDAC, this study not only introduces a compelling strategy for targeting GSK3 and MEK pathways but also signifies a leap toward a more effective treatment paradigm. With these findings, the horizon of hope expands for more tailored and impactful therapeutic interventions against this challenging malignancy.

https://onlinelibrary.wiley.com/doi/full/10.1111/CAS.16100

